# Mapping dental biofilms: from plaque index through planimetry to volumetric analysis

**DOI:** 10.1007/s00784-025-06729-z

**Published:** 2026-01-05

**Authors:** Katja Povšič, Luka Fijavž, Haris Munjaković, Adrian Kašaj, Rok Gašperšič

**Affiliations:** 1 Department of Oral Medicine and Periodontology – Dental Clinic, University Medical Centre Ljubljana, Hrvatski trg 6, Ljubljana, 1000 Slovenia; 2 Department of Oral medicine and Periodontology, Faculty of Medicine, University of Ljubljana, Ljubljana, Slovenia; 3 Department of Periodontology and Operative Dentistry, University Medical Centre Mainz, Mainz, Germany

**Keywords:** Dental plaque, Dental deposits, Dental plaque index, 3 d imaging, Diagnostic imaging, Computer assisted image analysis

## Abstract

**Objectives:**

Conventional plaque assessment methods, such as clinical indices and planimetry, rely on plaque-disclosing agents and may overemphasize thin biofilm areas due to plaque thickness variations. This study introduces a digital 3D method to quantify and visualize dental plaque volume from consecutive intraoral scans (IOS) and compares it with the Turesky Modification of the Quigley-Hein Plaque Index (TMQHPlI) and planimetry.

**Methods:**

After professional supragingival debridement at baseline (T0), fifteen participants refrained from oral hygiene for four days (T4). De novo plaque formation was assessed at T4 using TMQHPlI after application of a two-tone (pink/purple) plaque-disclosing agent. IOSs (3Shape TRIOS 4) were obtained at T0 and T4. Plaque accumulation was quantified using color-coded IOS comparisons with the Volumetric Plaque Index (VPI) and Adjusted VPI (AVPI). Machine learning (Trainable Weka Segmentation) was used to calculate the Planimetric Plaque Index (PPI).

**Results:**

A correlation between plaque surface area and volume was observed only for purple/mature plaque (*p* = 0.043). Early plaque growth was dominated by expansion of pink-stained, newly formed plaque. Once more than one-third of the surface was covered, further increases resulted mainly from vertical thickening rather than lateral spread.

**Conclusions:**

3D volumetric analysis offers a comprehensive, objective, and clinician-friendly method for assessing dental biofilms.

**Clinical relevance:**

The proposed 3D approach enables accurate monitoring of plaque accumulation and maturation, improving personalized oral hygiene assessment, patient education, and clinical decision-making.

**Supplementary Information:**

The online version contains supplementary material available at 10.1007/s00784-025-06729-z.

## Introduction

Dental biofilms are complex microbial communities on tooth-surfaces [1]. Their accumulation leads to dental caries and periodontitis [2]. Accurate assessment and monitoring of dental biofilms is essential for preventing biofilm-associated diseases. Their systematic detection and quantification is critical for optimizing therapeutic and preventive strategies aimed at managing biofilm-related oral pathologies [3].

Dental biofilm deposits are commonly assessed with traditional plaque indices (TPIs) using plaque-disclosing agents [4]. Despite being the gold standard for dental biofilm evaluation, their diagnostic accuracy is compromised by intra- and inter-examiner variability, subjectivity, and diminished precision at low/high plaque levels [5]. Because of their categorical nature and inconsistent inter-score intervals, TPIs also fail to detect potentially relevant changes in dental biofilm quantity [6].

To overcome this, image-based, computer-assisted approaches to intraoral scans (IOSs) and/or conventional photographs have been used to planimetrically evaluate dental biofilms [5]. Planimetry typically relies on plaque-disclosing agents to visualize plaque, enabling the calculation of plaque-covered tooth-surfaces, expressed as the Plaque Percentage Index (PPI) [7]. Alternatively, planimetry can also use fluorescence imaging, leveraging Quantitative Light-Induced Fluorescence and bacterial autofluorescence enhanced by a dye, to noninvasively detect dental biofilms [8]. Raw data is processed by image-analysis software, which enhances the contrast of plaque and performs tooth detection, segmentation, and plaque scoring using semi-automated [6, 9, 10] algorithms or machine-learning [11–13]. Nevertheless, all planimetric approaches rely on 2D-image analyses, even when using 3D-data from IOSs to capture plaque [14].

A novel approach for the analyses of IOSs based on the quantification of plaque volume using superimposed sequential 3D-digital models (e.g. pre- and post-plaque removal) has recently been proposed [15]. The method enables the visualization of differences in tooth-surface topography between model pairs, presented as color-coded maps, that illustrate the distribution of plaque thickness across tooth-surfaces.

The aim of this study was to investigate the relationship between the Turesky Modification of the Quigley Hein Plaque Index (TMQHPlI [16, 17]), 2D-planimetric and 3D-volumetric assessment of dental biofilms, captured with a IOSs. The TMQHPlI, a TPI, served as the reference.

## Materials and methods

The study took place from January–February 2023 at the Dental Clinic of the University Clinical Centre in Ljubljana, Slovenia. It was registered on ClinicalTrials.gov (NCT05709015) and approved by the National Medical Ethics Committee of Slovenia (0120–444/2022/3). Written informed consent was obtained from all participants. The study adhered to the Declaration of Helsinki and STROBE protocol. It was financed by the Ministry of Higher Education, Science and Innovation, Republic of Slovenia (Grant no.: P3-0293).

### Design

**Fig. 1:**
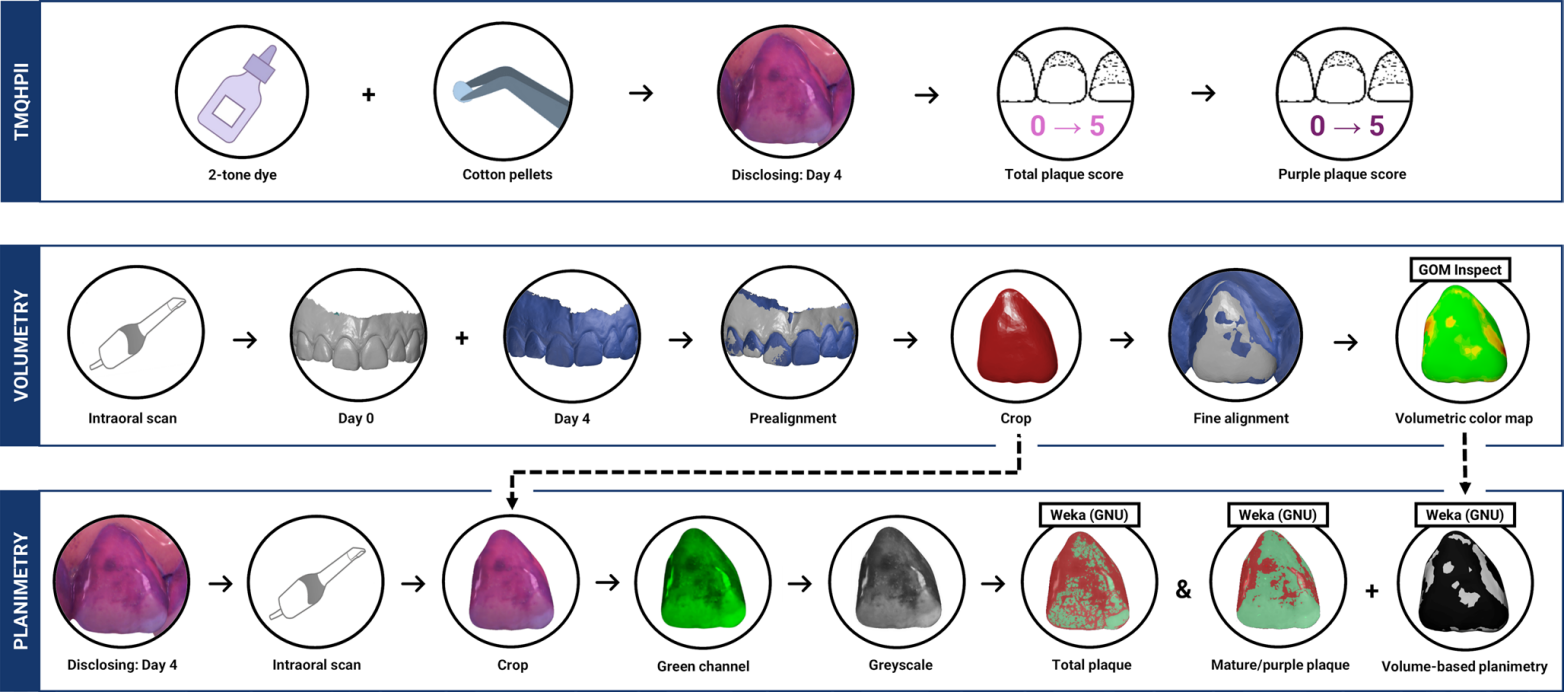
Workflow of dental plaque assessment using Turesky’s modification of Quigley Hein Plaque index, planimetry and volumetry. Note. TMQHPlI: Turesky Modification of the Quigley-Hein Plaque Index

This was a 4-day non-brushing plaque-accumulation study, originally described by Addy et al. [18]. Volunteers were periodontally-healthy individuals who refrained from all mechanical/chemical plaque control throughout the study. Clinical examinations and IOSs were performed at the beginning (T0) and end (T4) of the study by K.P. to assess plaque clinically, planimetrically and volumetrically (Fig. 1). Inclusion/exclusion criteria are presented in Supplementary Table 1.

### Clinical protocol

The full protocol has been described previously [15]. In short, participants received supragingival prophylaxis, polishing, and flossing to remove all calculus, plaque, and stains, followed by intraoral scanning (Trios 4; 3Shape, Copenhagen, Denmark) at T0. Moisture was cleared from the teeth with an air syringe before and during scanning, and the buccal/labial mucosa was held aside with mouth mirrors. Subjects then abstained from mechanical/chemical oral hygiene practices for 4-days. At T4, new IOSs were performed, and a two-tone plaque-disclosing agent (dyes: CI45410, CI42090) was applied (Curaprox PlaqueFinder-260; Curaden, Kriens, Switzerland) using cotton pellets. Plaque was assessed on three sites (mesial, central, distal) per each buccal tooth-surface of all incisors, canines and premolars using the TMQHPlI for both dark-disclosed (DDP; blue/purple staining; supposedly mature/old plaque) and total-disclosed plaque (TDP; pink + blue/purple staining; supposedly new plaque) according to Volgenant et al. [19]. The calibration of the examiner (K. P.) has been described previously, demonstrating a Cohen’s kappa of 0.82 [15]. The study ended with a final prophylaxis and fluoride treatment.

### Volumetric plaque analysis

The volumetric plaque analysis was conducted by K. P. using GOM Inspect 2022 (GOM GmbH, Braunschweig, Germany), as described previously [15] (Supplementary Fig. 1). The digital workflow included: digital model acquisition at T0 and T4 from IOSs; pre-alignment of T0/T4 models [20]; computer-assisted delineation of buccal tooth-surface margins (i.e. regions of interest: ROIs); best-fit superimpositions of T0/T4 ROI pairs (Supplementary Fig. 1); visualization of topographic changes between each T0/T4 ROI pair using colour-coded maps based on the thickness of *de-novo* formed plaque; and volumetric assessment of plaque using the volumetric plaque index (VPI) and adjusted volumetric plaque index (AVPI). The calibration exercise has been described previously, demonstrating that the ROI average mean absolute distance was 0.0099 mm (SD = 0.0028 mm) [15].

### Planimetric plaque analysis

Planimetric plaque analysis was performed by L. F. following two separate protocols: one for the planimetric analysis of disclosed plaque and one for the planimetric analysis of volumetric colour-maps.

#### Planimetric analysis of disclosed plaque

Screenshots of each disclosed buccal tooth-surface were captured from IOSs using the same projection as in the volumetric workflow; ROIs were applied as masks in Adobe Photoshop v.20.0 (Adobe Inc., San Jose, CA, USA) to crop each surface. The green channel of each image was isolated for optimal plaque contrast and converted to grayscale images for segmentation in ImageJ v.2.16.0 (NIH, Bethesda, MD, USA) using the Trainable Weka Segmentation plugin [21]. The two-tone plaque-disclosing agent allowed separate assessment of dark/purple (PLANIdark) and total (PLANItot) plaque, with light/pink (PLANIlight) plaque calculated as PLANIlight = PLANItot – PLANIdark (Supplementary Fig. 2).

#### Planimetric analysis of volumetric colour maps

Colour-coded maps showing plaque thickness changes between T0/T4 ROI pairs were converted into black-and-white Boolean images. A 0.01 mm cut-off was used to distinguish plaque-present (white) from plaque-absent (black) areas, reflecting measurement precision at the level of the VPI’s limit of detection, as previously described [15]. Screenshots of each map, captured in the same projection as the volumetric workflow, were analysed planimetrically (Supplementary Fig. 3) for total plaque coverage (PLANIvolmap) using the same segmentation method described in Section "Planimetric analysis of disclosed plaque".

### Sample size

Based on a previous study on the volumetric evaluation of dental plaque, sample size was calculated using the correlation between the VPI and TMQHPlI scores for TDP (*r* = 0.66 at α = 0.05, β = 0.2) and was found to be 15 subjects [15]. Sample size was increased by 10% to account for drop-outs.

### Statistical analysis

Planimetric and volumetric outcomes were described with means and standard deviations (SD). The Shapiro-Wilk test was used to assess normality of distribution. Mean TMQHPlI scores were presented as averages of three scores per tooth-surface. Comparisons between PLANI-tot, PLANIdark, PLANIlight and PLANIvolmap was performed with Friedman tests; pairwise post-hoc analyses were calculated using Wilcoxon Signed-Rank Tests with Bonferroni corrections. Spearman’s rho and Passing-Bablok regressions were used to calculate the relationship between the mean TMQHPlI, planimetric and volumetric outcomes; all values were transformed into z-scores for the Passing-Bablok analyses. The distributions of PLANIdark and PLANIllight were modelled with linear functions; a colour legend based on relative planimetric values of both variables in relation to relPLANItot (where PLANItot = 100%) was used: relPLANIdark (relative planimetric values for DDP; i.e. PLANIdark/PLANItot×100) and relPLANIlight (relative planimetric values for LDP; i.e. PLANIlight/PLANItot×100). For standardization, only buccal surfaces of maxillary premolars, canines and incisors were used for statistical analysis. To evaluate the diagnostic performance of the VPI against planimetry, Receiver Operating Characteristic (ROC) curve analysis was performed. The planimetric values were dichotomized into binary categories (10:90%, 25:75% and 50:50% plaque coverage); the VPI was used as the test variable. The area under the ROC curve (AUC) was plotted. All analyses were performed in SPSS v. 26 (IBM, San Jose, California, USA).

## Results

### Study population

Sixteen volunteers were included; one withdrew on day-3 for personal reasons. The final sample included 15 individuals (7 males, 8 females; mean age: 25 years). One participant had a retained deciduous upper right second molar, which was excluded from the analysis. Another participant presented with congenital aplasia of both maxillary lateral incisors, which were also excluded from statistical evaluation. In total, 147 tooth-surfaces were analysed; none had buccal restorations.

### Clinical, planimetric and volumetric evaluations of plaque

Descriptive statistics of clinical, planimetric and volumetric indexes at T4 are presented in Table 1. On average, TDP covered one-third of the analysed tooth-surfaces, while DDP was present either in the form of flecks or a thin continuous band at the gingival crevice.

**Table 1:** Descriptive statistics of the clinical, planimetric and volumetric plaque indexes 4 days after the cessation of all mechanical and chemical oral hygiene practices

	Mean ± SD	Range (min–max)	*p*-value
TDP TMQHPlI	2.59 ± 0.93	1–5	**< 0.001** ^2^
DDP TMQHPlI	1.42 ± 0.87	0–3	
PLANItot (%)	45.80 ± 18.21	4.03–96.33	**< 0.001** ^1^
PLANIdark (%)	24.25 ± 14.41	1.66–82.04	
PLANIlight (%)	21.54 ± 13.21	0.77–63.72	
PLANIvolmap (%)	18.57 ± 8.94	1.20–42.82	
VPI (mm^3^)	0.90 ± 0.36	0.28–2.00	**< 0.001** ^2^
AVPI (mm^3^/mm^2^)	0.02 ± 0.01	0.01–0.03	

*PLANItot* planimetric evaluation of all disclosed plaque per tooth-surface area, *PLANIdark* planimetric evaluation of dark/purple disclosed plaque per tooth-surface area, *PLANIlight* planimetric evaluation of light/pink disclosed plaque per tooth-surface area, *PLANIvolmap* planimetric evaluation of plaque per tooth-surface area based on binary (yes/no) volumetric colour map with cutoff 0.01 mm; *VPI* volumetric plaque index, *AVPI* adjusted volumetric plaque index, *DDP TMQHPlI* Turesky’s modification of Quigley Hein Plaque index for dark disclosed plaque; *TDP TMQHPlI* Turesky’s modification of Quigley Hein Plaque index for total disclosed plaque, *SD* standard deviation, *min* minimal value, *max* maximal value; bold: statistical significance at α = 0.05; ^1^: Friedman test; ^2^: Wilcoxon signed rank test

In terms of planimetry, the average ratio of DDP vs. LDP per plaque-covered tooth-surface area was approximately 1:1. When combined as PLANItot, they covered about half (45%±18%) of each tooth-surface on average. A notable discrepancy was observed between the planimetric assessment of IOS screenshots and volumetric colour maps: PLANItot was, on average, found to be 2.47 times larger than PLANIvolmap and correspond best to PLANIdark values. *Post-hoc* tests showed statistically-significant differences between PLANItot–PLANIdark (*p* < 0.001), PLANItot–PLANIlight (*p* < 0.001), PLANItot–PLANIvolmap (*p* < 0.001) and PLANIdark–PLANIvolmap (*p* < 0.001).

The volumetric analysis revealed that after 4-days of non-brushing, plaque volume per tooth-surface peaked at 2.00 mm³, the average VPI measuring approximately 1.00 mm³.

### Relationship between clinical, planimetric and volumetric plaque indexes

**Fig. 2:**
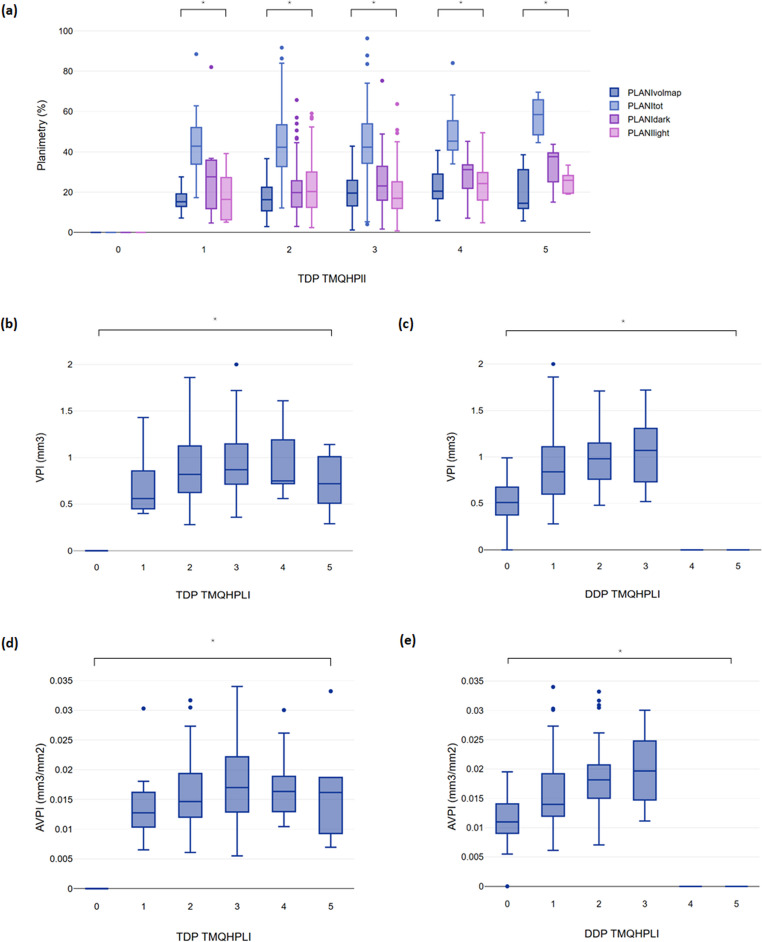
Agreement between the clinical, planimetric and volumetric assessments of dental plaque. Relationships between the planimetric assessment and TMQHPlI scores for TDP (**a**). Relationships between the volumetric assessment (VPI) and TMQHPlI scores for TDP (**b**) and DDP (**c**). Relationships between the weighted volumetric assessment (AVPI) and TMQHPlI scores for TDP (**d**) and DDP (**e**). Note. PLANItot: planimetric evaluation of all disclosed plaque per tooth-surface area; PLANIdark: planimetric evaluation of dark/purple disclosed plaque per tooth-surface area; PLANIlight: planimetric evaluation of light/pink disclosed plaque per tooth-surface area; PLANIvolmap: planimetric evaluation of plaque per tooth-surface area based on binary (yes/no) volumetric colour map with cutoff 0.01 mm; VPI: volumetric plaque index; AVPI: adjusted volumetric plaque index; TDP TMQHPlI: Turesky’s modification of Quigley Hein Plaque Index for dark disclosed plaque; TDP TMQHPlI: Turesky’s modification of Quigley Hein Plaque Index for total disclosed plaque

The distributions of the volumetric and planimetric measurements according to the mean TMQHPlI score per tooth-surface (average of 3-scores) rounded to the nearest whole number are presented in Fig. 2. The values of the different planimetric approaches differed significantly for each TMQHPlI value at the level of both DDP (not shown) and TDP (*p* < 0.001) (Fig. 2a). In general, total planimetric coverage increased with increasing TMQHPlI values: at scores 3–5, this trend occurred mainly due to increases in DDP. The VPI and AVPI distributions differed significantly between TMQHPlI scores at the level of both DDP (Fig. 2c, e) and TDP (Fig. 2b, d) (*p* < 0.001). DDP never exceeded TMQHPlI = 3, with steady increases in the VPI and AVPI up to this score. TDP encompassed all TMQHPlI scores from 1 to 5 on account of pink-coloured plaque. The highest volume of TDP, estimated either by the VPI or AVPI, was at TMQHPlI = 3, decreasing at both lower and higher TMQHPlI values. DDP never exceeded one-third of any tooth-surface (no TMQHPlI scores 4–5). This indicates that the greatest total plaque volume was found when mature plaque (DDP) covered between one- and two-thirds of a tooth-surface (TMQHPlI = 3).

**Fig. 3:**
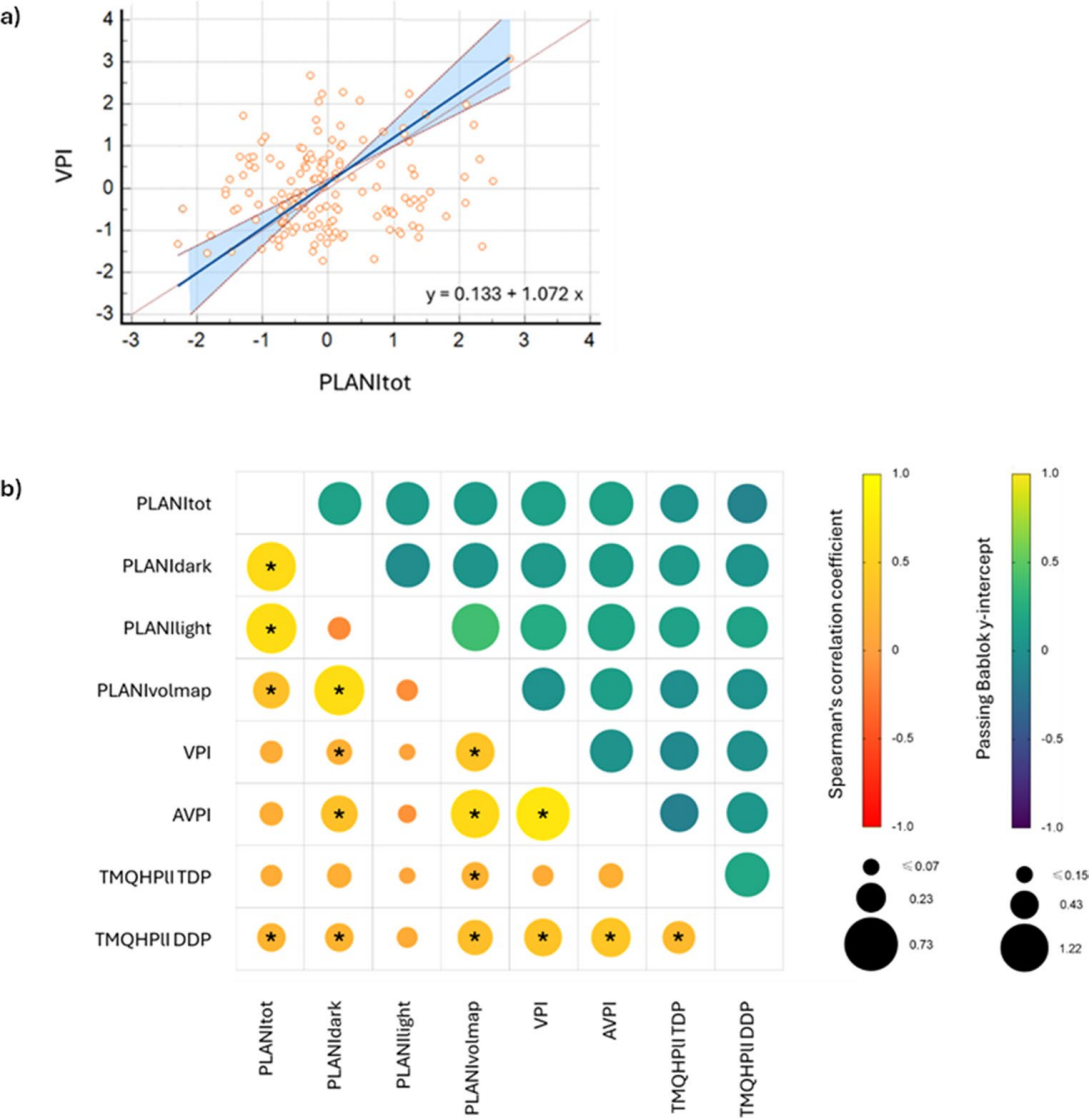
Relationship between clinical, planimetric and volumetric plaque indexes: (**a**) Passing Bablok regression of VPI vs. PLANItot; (**b**) Spearman’s correlation and Passing Bablok matrix. Note. PLANItot: planimetric evaluation of all disclosed plaque per tooth-surface area; PLANIdark: planimetric evaluation of dark/purple disclosed plaque per tooth-surface area; PLANIlight: planimetric evaluation of light/pink disclosed plaque per tooth-surface area; PLANIvolmap: planimetric evaluation of plaque per tooth-surface area based on binary (yes/no) volumetric colour map with cutoff 0.01 mm; VPI: volumetric plaque index; AVPI: adjusted volumetric plaque index; TMQHPlI DDP: Turesky’s modification of Quigley Hein Plaque index for dark disclosed plaque; TMQHPlI TDP: Turesky’s modification of Quigley Hein Plaque index for total disclosed plaque; *: statistical significance at α = 0.05

The relationships between clinical, planimetric and volumetric variables are shown in Fig. 3. While significant correlations were found between PLANItot, PLANIdark, PLANIlight and PLANIvolmap, the relationship between the volumetric and planimetric assessment of plaque was statistically-significant (*p* = 0.043) only at the level of DDP (i.e. mature plaque). Apart from PLANIlight, TMQHPlI scores correlated with all plaque measures. The Passing-Bablok regression analyses showed scattered data with no apparent trend at the level of any index pair. Nevertheless, no significant proportional or constant bias was found between the clinical, planimetric and volumetric indices, indicating good agreement.

**Fig. 4:**
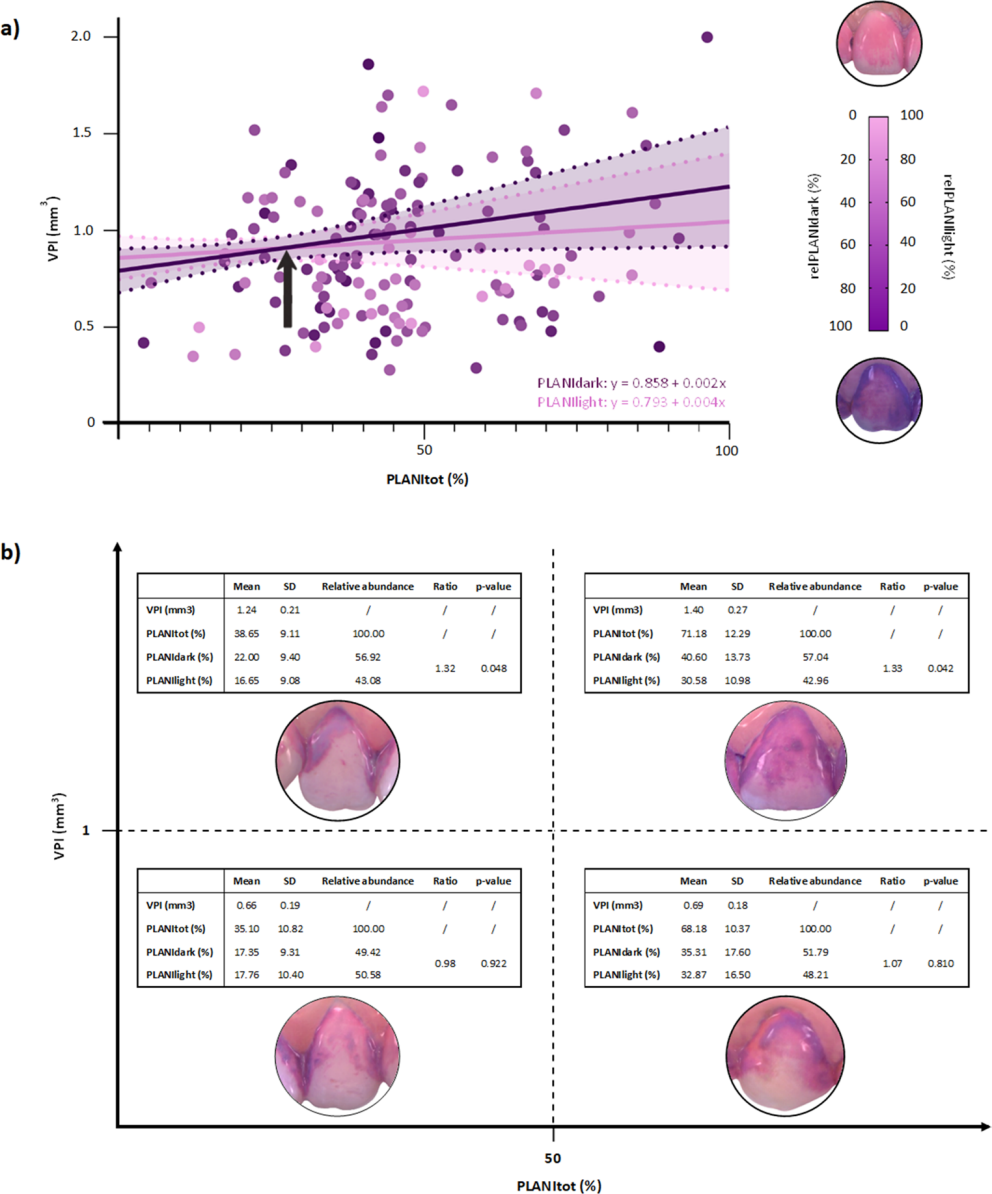
Planimetric vs. volumetric plaque assessment. (**a**) Relationship between dental plaque volume and planimetry. (**b**) Ratios of planimetric variables and their contributions to dental plaque volume. Note: PLANItot: planimetric evaluation of all disclosed plaque per tooth-surface area; relPLANIdark: relative planimetric evaluation of dark/purple disclosed plaque per tooth-surface area (i.e. PLANIdark/PLANItot*100); relPLANIlight: relative planimetric evaluation of light/pink disclosed plaque per tooth-surface area (i.e. PLANIlight/PLANItot*100); VPI: volumetric plaque index; SD: standard deviation; →: in Fig. 4a, when dental plaque covered more than 32.5% of a tooth-surface, increases in plaque volume were mainly driven by mature plaque, with the VPI rising twice as much per unit of surface area in the presence of mature compared to newly formed plaque

The two-tone plaque-disclosing agent showed that increases in the surface-area/planimetry of mature plaque contributed more to total plaque volume than increases in the surface-area of newly-formed plaque; the latter caused minimal overall increases (Fig. 4a). When newly-formed plaque covered < 32.5% of the surface-area, its contribution to plaque volume tended to be higher than that of mature plaque. Beyond 32.5%, increases in VPI were mainly driven by mature plaque, with the VPI rising twice as much per unit of surface-area in the presence of mature compared to newly-formed plaque. In other words: when <⅓ of a tooth-surface was covered with plaque, i.e. when plaque accumulation was in its early stages, newly-formed plaque deposits contributed most to plaque volume; however, once >⅓ of a tooth-surface was covered, plaque began to thicken (and volume began to increase) mostly due to the maturation of existing dental plaque.

On tooth-surface areas with VPI > 1mm^3^, the ratios between average relPLANIdark and average relPLANIlight measurements were ≈ 1.33. When the VPI measured < 1mm^3^, the ratios were ≈ 1.00 (Fig. 4b). It was once again found that increases in the VPI after initial plaque accumulation were mainly attributed to deposits of DDP. The results of the ROC curve analysis are presented in Supplementary Fig. 4.

## Discussion

This study compared 2D-planimetric, 3D-volumetric and traditional clinical approaches to dental plaque evaluation. A statistically-significant relationship between volumetric and planimetric plaque scores was found only for mature plaque (DDP – dark/purple disclosed plaque). At the early stages of plaque accumulation, when <⅓ of tooth-surface areas were covered, pink-stained/newly-formed plaque contributed most to total plaque volume. However, as plaque coverage exceeded one-third of a tooth-surface, the accumulation pattern shifted: plaque began to increase in thickness (vertical growth) rather than with surface-area (horizontal growth). In these more advanced stages of plaque accumulation, volumetric increases were driven mainly by the maturation of existing/purple plaque and not by the expansion of pink/newly-formed plaque, keeping its surface-area largely unchanged. These observations are based on a sample of young, periodontally healthy adults, focusing solely on maxillary buccal surfaces of anterior and premolar teeth, and the relatively small sample size should be considered when interpreting the results. The upper limit of plaque thickness over time remains to be determined.

TPIs [17, 22, 23] assess disclosed plaque on tooth-surface regions. Even though they remain the golden standard, their diagnostic accuracy is limited by intra-/inter-examiner variability, inherent subjectivity and robustness at low/high plaque scores. Additionally, unequal increments between scores hinder statistical analyses since the assumption of a constant marginal effect is violated, leading to ambiguous/misleading interpretations [5]. Plaque changes, as measured by TPIs, can obscure the actual extent of plaque removal/accumulation, since complex plaque distribution patterns are reduced to simple index values. Even though statistically-significant differences may be observed, their magnitude is distorted and the changes are often too small for clinical relevance/interpretation [24]. In the present study, plaque measurements were analysed as independent, non-nested observations, potentially underestimating variance, leading to underestimated standard errors and inflated Type I error rates.

Plaque indexes based on numerical (PPI, VPI) rather than ordinal scales have resolved this challenge. However, these approaches also have limitations. Planimetry is performed on 2D-projection images, even when IOSs are used to acquire 3D-data of plaque [14]. Plaque area is therefore accurately measured only in zones that are perpendicular to the optical axis of the camera and is significantly underestimated with increasing angles of deviation (interdentally, posteriorly). Since tooth curvature (mesially/distally) isn’t considered, distortion errors resembling those found on world map projections (e.g. orthographic projection) occur at the edges of each 2D-image. This error is amplified when cameras are used without standardized set-ups since the repeatability of a photograph’s projection (i.e. the angle of the camera with reference to the tooth-surface) is never 100% (e.g. like used by Del Rey et al. 2023). This error doesn’t occur when using 3D-data from IOSs.

Planimetry can be performed using semi-automated methods or machine-learning. Semi-automated techniques, typically based on fluorescence imaging and basic image processing, improve reproducibility over manual scoring but still rely on operator input and are limited in scalability [9]. In contrast, trainable machine-learning software automates the detection and segmentation of plaque and teeth, reducing subjectivity and enabling robust analyses. Machine-learning is particularly advantageous in complex imaging conditions, such as variations in lighting, angle, or scattered plaque distribution, where rule-based approaches may fail. Studies using convolutional neural networks such as DeepPlaq [12, 13] have shown that machine-learning based planimetry achieves high accuracy, with intersection-over-union (IoU) scores above 0.75 and improved consistency across diverse datasets. While machine-learning models require annotated data (e.g. delineation of tooth-surfaces and plaque boundaries for deep learning) and careful validation, they offer superior scalability, objectivity, and potential for real-time clinical application.

Planimetry is fundamentally dichotomous: it describes each surface point as positive (plaque-present) or negative (plaque-absent). However, the limit of plaque detection, i.e. minimum plaque thickness at the boundary between ‘plaque-covered’ and ‘plaque-free’ areas, remains undetermined. Unlike the VPI and AVPI, the PPI cannot measure absolute amounts of plaque, which vary across tooth-surfaces due to anatomical heterogeneities (deep/shallow grooves, lingulae, valleys, gingival margins). This is important because the thinnest layer of plaque contributes the least to total plaque volume but the most to total surface-area coverage; an inverse relationship therefore appears between the plaque-covered tooth-area and the average plaque thickness. This may explain the lack of a correlation between total plaque volume and total plaque surface-area and suggests that they don’t measure the same entity. Indeed, the PPI is a measure of surface-area/plaque distribution, while the VPI quantifies plaque based on its thickness.

The two-tone plaque-disclosing agent used in the present study had a particular advantage: it distinguished pink/newly-formed biofilms from purple/mature ones [19]. Differential staining occurs due to differences in pH and thickness; pink dye adheres to all plaque, whereas the blue component adheres and diffuses into denser/thicker plaque [19]. According to our findings, newly-formed plaque generally covers the surface of the tooth in a thin layer. However, there is no clearly defined plaque thickness cut-off which would determine whether plaque will stain purple/pink. It is therefore possible that colour-based (pink/purple) thresholds don’t match plaque thickness thresholds. Further research is needed to determine the exact binding sites of each dye to the chemical elements of biofilms and precisely define the thickness thresholds of pink/purple stained plaque. This would allow a direct comparison of different dyes.

Planimetry performed on images/screenshots of IOSs based on staining with plaque-disclosing agents have several drawbacks. Since IOSs are intended for capturing geometry rather than accurate colour, the resulting images can misrepresent certain hues, especially subtle or blended ones like purple. Colour shade detection by IOSs may additionally be influenced by external factors: light, tooth-texture and scanner manipulation. The accuracy of colour-shade matching for TRIOS 3 (i.e. how close measurements are to their true value) has been assessed by comparing provided readings against reference values and found to measure from 43.9 to 66% [25]. Colour visualisation may also be influenced by intrinsic software settings (contrast/brightness/shadows) and graphical properties of computer screens [26].

Volumetric approaches, on the other hand, overcome these limitations since they facilitate accurate measurements of tooth-surface areas, eliminate issues related to projection repeatability, and are derived directly from superimposed IOSs, avoiding the need for staining. Additionally, they provide visualization of plaque thickness using colour-coded maps, which display either the exact thickness at each point using a continuous colour scale or categorized areas of similar thickness based on clinically relevant thresholds. Since plaque thickness is analysed point-to-plane, the detection limit is much lower (≈ 10 μm) than that of planimetric methods or TPIs [15]. This is clearly reflected in areas where the visual assessment of plaque index is negative (TMQHPlI = 0), but the volumetric analysis indicates a certain plaque volume. The plaque detection limit is an intrinsic property of the current available technology and will undoubtedly be overcome in the future through technological advancements and computational methods. Additional limitations of the VPI include inaccuracies arising from scanning distortions. These typically occur in posterior/curved regions due to limited access, large scanner tip size, movement during scanning, and challenges in 3D-model reconstructions [27, 28]. The accuracy of superimpositions additionally depends on the algorithms of the selected software used in the process. By integrating the workflow into automated IOS software, it has the potential to become clinician-friendly by minimizing manual steps.

## Conclusion

Since planimetry and volumetry assess inherently different properties of plaque, i.e. surface-area vs. thickness, a statistically-significant correlation between the VPI and PPI was observed only at the level of mature plaque (i.e., DDP, dark/purple disclosed plaque). In the more advanced stages of plaque accumulation, increases in volume primarily occurred due to the maturation of existing plaque deposits, rather than the spread of newly formed plaque.

## Supplementary Information

Below is the link to the electronic supplementary material.

Supplementary Material 1

## Data Availability

The original data is available in the following data repository: Povšič, Katja; Munjaković, Haris; Fidler, Aleš; Gašperšič, Rok (2024). 3D intraoral scans - de novo plaque formation. figshare. Dataset. https://doi.org/10.6084/m9.figshare.25768860.v1.
