# Supplementary material for: Mapping dental biofilms: from plaque index through planimetry to volumetric analysis
Source: Clin Oral Investig. 2026 Jan 5;30(1):38. doi: 10.1007/s00784-025-06729-z (PMC12765721; doi:10.1007/s00784-025-06729-z)
Supplement: Supplementary file 1 — Supplementary Material 1 [file 784_2025_6729_MOESM1_ESM.docx]

**SUPPLEMENTAL APPENDIX**

**Mapping dental biofilms: from plaque index through planimetry to volumetric analysis**

Katja Povšič^1,2^, Luka Fijavž^2^, Haris Munjaković^1,2^, Adrian Kašaj^3^, Rok Gašperšič^1,2^

^1^ Department of Oral Medicine and Periodontology, University Medical Centre Ljubljana, Ljubljana, Slovenia

^2^ Department of Oral medicine and Periodontology, Faculty of Medicine, University of Ljubljana, Ljubljana, Slovenia

^3^ Department of Periodontology and Operative Dentistry, University Medical Centre Mainz, Mainz, Germany

**Corresponding author:** Katja Povšič, Department of Oral Medicine and Periodontology, Faculty of Medicine, University of Ljubljana, Hrvatski trg 6, 1000 Ljubljana, Slovenia; telephone: 0038615224889 (katja.povsic@kclj.si).

**Supplementary table 1.** Inclusion and exclusion criteria.

| **Inclusion criteria** | **Exclusion criteria** |
| --- | --- |
| - Age 20-30 years - Non-smokers - Systemically healthy - No regular systemic medication - Presence of at least 6 teeth in each jaw quadrant - Plaque index < 10 % at baseline - No periodontal pockets measuring > 4 mm | - Gingivitis - Regular use of a mouthwash as part of oral hygiene routine - Antibiotic treatment in the last 6 months - Pregnancy - Lactation - Fixed/removable prosthetic devices - Dental implants - Systemic illness (i.e. HIV/AIDS, diabetes mellitus, cancer, bone metabolism diseases, diseases affecting wound healing) - Treatment with immunosuppressive therapy, chemotherapy, radiation therapy, calcium antagonists, antiepileptics, non-steroidal anti-inflammatory drugs |

**Supplementary figure 1.** Workflow of volumetric plaque evaluation.

The 3D model analyses were conducted in GOM Inspect 2022 (GOM GmbH, Braunschweig, Germany). After importing the baseline (T0) and follow-up (T4) datasets into the software, an initial global alignment was performed. This step, required for any subsequent superimposition, automatically matched the full morphology of both scans—including teeth and surrounding soft tissues—using the using the iterative closest point (ICP) procedure (Besl and McKay, 1992).

Regions of interest (ROIs), corresponding to the vestibular and oral surfaces of each tooth, were then defined on the T0 models. To delineate these surfaces, a boundary curve was manually traced with the Surface Curve tool. The curve was generated using the Maximum Curvature function, placing approximately 5–10 snap-to points along the gingival margin and the incisal/occlusal border (Kuralt & Fidler, 2022). The software subsequently identified the mesial and distal limits of each ROI by automatically detecting areas of maximum surface curvature, which were then connected to the manually created gingival and incisal/occlusal curves.

Each T0 ROI was individually matched to its corresponding tooth surface on the T4 model using the Local Best-Fit function (Kuralt & Fidler, 2021). For every alignment, the deviation was quantified as the mean absolute distance between the mesh points and the defined CAD surface (Vág et al., 2019).

Surface changes between the T0 and T4 ROIs were visualised via the Surface Comparison tool. Colour-coded deviation maps representing the topographical differences between paired surfaces were used to compute the volume of material accumulated on each tooth, expressed as the volumetric plaque index (VPI, mm³). Because tooth morphology varies, plaque volume was normalised to the ROI surface area (vestibular/oral area) to yield the adjusted volumetric plaque index (AVPI), reported in mm³/mm².

Besl, P. J., & McKay, N. D. (1992). A Method for Registration of 3-D Shapes. *IEEE Transactions on Pattern Analysis and Machine Intelligence*, *14*(2), 239–256. https://doi.org/10.1109/34.121791

Kuralt, M., & Fidler, A. (2021). Assessment of reference areas for superimposition of serial 3D models of patients with advanced periodontitis for volumetric soft tissue evaluation. *Journal of Clinical Periodontology*, *48*(6), 765–773. https://doi.org/10.1111/JCPE.13445

Kuralt, M., & Fidler, A. (2022). A novel computer-aided method for direct measurements and visualization of gingival margin changes. *Journal of Clinical Periodontology*, *49*(2), 153–163. https://doi.org/10.1111/JCPE.13573

*Povšič K, Munjaković H, Erčulj V, Aleš Fidler, Rok Gašperšič. 2025. 3D Method for the Volumetric Evaluation and Visualisation of Dental Biofilms: A Proof-of-Principle Study. J Clin Periodontol. 0:1–11. doi:10.1111/JCPE.70019.*

Vág, J., Nagy, Z., Simon, B., Mikolicz, Á., Kövér, E., Mennito, A. S., Evans, Z. P., & Renné, W. G. (2019). A novel method for complex three-dimensional evaluation of intraoral scanner accuracy. *International Journal of Computerized Dentistry*, *22*(3), 239–249.

**Supplementary figure 2.** Planimetric analysis of disclosed plaque.

A screenshot of each disclosed buccal tooth surface was obtained in the same projection used for the delineation of ROIs in the volumetric workflow described above. The ROI was then used as a mask to crop the exact shape of each buccal tooth surface on the obtained 2D-screenshot using Adobe Photoshop v. 20.0 (Adobe Inc., San Jose, California, USA). Since each coloured pixel (excluding black) of the ROI contained red/green/blue components, (Jung et al. 2022) the thresholds for red and blue were set to zero. This isolated the green channel, which provided the highest contrast against the red tones of the PDA and visually enhanced the stained plaque. Each ROI was then converted to grayscale based on the intensity values of the green channel. Next, classifier training for machine-learning segmentation and final segmentation without further manual corrections of greyscale differences was performed using the Trainable Weka Segmentation plugin (Arganda-Carreras et al. 2017) (Howard Hughes Medical Institute, Janelia Research Campus, Ashburn, Virginia, USA) in ImageJ v. 2.16.0 (National Institutes of Health, Bethesda, Maryland, USA).

The two-tone PDA enabled the independent planimetric assessment of dark/purple-coloured tooth surface-areas (PLANIdark), and light/pink-coloured tooth surface-areas (PLANIlight). Accordingly, the classifier for segmentation was separately trained for PLANIdark and PLANIlight, respectively. The threshold was visually examined directly within the program. Due to the continuous spectrum of colour intensity observed in plaque deposits, light/pink-disclosed plaque (LDP) could not be distinguished from DDP and white/plaque-free surfaces simultaneously; PLANIlight was therefore calculated from the total plaque covered surface-area (PLANItot) using the following equation:

PLANItot = PLANIdark + PLANIlight

PLANIlight = PLANItot – PLANIdark

*Jung K, Giese-Kraft K, Fischer M, Schulze K, Schlueter N, Ganss C. 2022. Visualization of dental plaque with a 3D-intraoral-scanner-A tool for whole mouth planimetry. PLoS One. 17(10). doi:10.1371/JOURNAL.PONE.0276686.*

*Arganda-Carreras I, Kaynig V, Rueden C, Eliceiri KW, Schindelin J, Cardona A, Seung HS. 2017. Trainable Weka Segmentation: a machine learning tool for microscopy pixel classification. Bioinformatics. 33(15):2424–2426. doi:10.1093/BIOINFORMATICS/BTX180.*

**Supplementary figure 3:** Planimetric analysis of volumetric colour maps

Colour-coded maps illustrating the thickness of newly formed dental plaque (i.e. changes in surface topography between each T0/T4 ROI pair) were used to generate black-and-white Boolean images of all the previously defined buccal tooth surface ROIs. The cut-off value between plaque-present/white and plaque-absent/black areas was set at a thickness of 0.01 mm; this value has previously been shown to approximate the average of the mean absolute distances across all ROIs at a full-mouth level and was a measure of measurement precision.(Povšič et al. 2025)

A screenshot of each Boolean volumetric colour map was obtained in the same projection used for the delineation of ROIs in the volumetric workflow (and was thus identical to the projection used for the planimetric evaluation of disclosed plaque). Planimetric evaluation of the total plaque covered surface based on the black-and-white colour maps (PLANIvolmap) was acquired by the same segmentation workflow as described in Section 2.4.1.

*Povšič K, Munjaković H, Erčulj V, Aleš Fidler, Rok Gašperšič. 2025. 3D Method for the Volumetric Evaluation and Visualisation of Dental Biofilms: A Proof-of-Principle Study. J Clin Periodontol. 0:1–11. doi:10.1111/JCPE.70019.*

**Supplementary figure 4.** Results of ROC curve analysis.

The ROC curve analysis using the VPI as the predictor showed a poor discriminative ability at higher planimetric thresholds, with an AUC of 0.535 (p = 0.502) for > 50 % plaque surface are threshold, and 0.546 (p = 0.548) for a > 25 % threshold, indicating no significant predictive value. At a lower threshold of > 10 %, the VPI’s AUC increased to 0.790, suggesting better discrimination for smaller plaque areas, although this was not statistically significant (p = 0.160). These findings suggest that the VPI may be more sensitive to detecting smaller areas of plaque.
